# Heterogeneity in the consumption of fresh and ultra-processed foods by the Brazilian population ≥10 years of age

**DOI:** 10.1590/1980-549720240069

**Published:** 2024-12-16

**Authors:** Victor Nogueira da Cruz Silveira, Ana Karina Teixeira da Cunha França, Alcione Miranda dos Santos

**Affiliations:** I Universidade Federal do Maranhão, Postgraduate Program in Colletive Health – São Luís (MA), Brazil.

**Keywords:** Processed food,, Diet, Brazil, Food consumption, Diet surveys

## Abstract

**Objective:**

To evaluate the heterogeneity in the consumption of fresh or minimally processed foods (FMPF) and ultra-processed foods (UPF) in the Brazilian population ≥10 years of age.

**Methods:**

Cross-sectional study that used data from the food consumption and resident module from the 2017–2018 edition of the Family Budget Survey. Variables relating to sex, region of residence, household status and per capita family income in minimum wages were used. The outcomes were dietary participation in percentage of FMPF and UPF. Heterogeneity was assessed using random effects produced by linear mixed-effects models.

**Results:**

Thirty-two random effects were obtained for the consumption of FMPF and 34 for UPF. Living in the urban area of the South and Southeast regions, as well as having a higher income were driving factors in the consumption of UPF and reducing the consumption of FMPF. Living in a rural area and having low income were mainly reducing factors in the consumption of UPF and driving factors in the consumption of FMPF.

**Conclusions:**

The consumption of UPF and FMPF was determined by the set of factors that represented easy access to these foods, whether geographic or economic such as income.

## INTRODUCTION

Fresh or minimally processed foods (FMPF) are those obtained directly from plants or animals that have not undergone any changes after leaving nature or have undergone minimal changes^1^. They are characterized by not containing chemical additives, thus becoming the basis for a healthy diet^2^. In contrast, ultra-processed foods (UPF) are industrial products made based on FMPF or synthesized in a laboratory, which have added chemical compounds that alter their sensory properties^1^.

These foods differ according to their degree of processing, as well as the addition of artificial substances^1–3^. The use of these industrial components in UPF guarantees a longer shelf life, palatability and some similarity with the FMPF to which they refer^1^.

The consumption of UPF has become a global concern, since their ingestion is associated with several harmful effects on health^4^. However, despite being recognized as harmful to health, their consumption shows an increasing trend, possibly due to the practicality and hyperpalatability that they offer^3,5^.

In Brazil, the consumption of UPF presents a diverse pattern, being higher among women, in the South and Southeast regions, as well as in populations with higher income and education, but with strong divergence regarding the classes of ultra-processed foods consumed^3,6,7^. Furthermore, despite the overall downward trend in the consumption of these foods, Brazil faces a trend of progression possibly caused by overt marketing and practicality in the consumption of these products^8^. In contrast, populations with lower purchasing power, racialized populations and those living in rural areas tend to consume less of these industrial products and have a diet rich in FMPF^3,8^.

Different studies have highlighted the sociodemographic effects on food consumption in Brazil, and usually use linear or generalized regression models to test their hypotheses^4^. However, food consumption is a multi-motivated behavior that can be modified by combining a set of factors, and not just one alone. In this sense, mixed-effects regression models emerge as powerful statistical tools to consider the variation between individuals and groups^9^.

When assessing the consumption of ultra-processed foods, these models can take into account individual factors — such as age, sex, education — and contextual factors — such as geographic region and income — to better understand the associations and predict changes over time. Thus, understanding the differences in the consumption patterns of ultra-processed foods is crucial for understanding and strategically targeting prevention and health promotion policies for a specific sector in order to mitigate the consumption of these foods, as well as prevent their harmful effects. By considering socioeconomic and demographic factors, specific interventions for higher-risk groups can be planned and implemented. Thus, this study aimed to evaluate the heterogeneity in the consumption of FMPF and UPF by the Brazilian population ≥10 years of age.

## METHODS

### Design and sample

A cross-sectional study was conducted using data from the personal food consumption module of the National Food Survey (INA) of the Household Budget Survey (POF) — a nationally representative survey conducted between July 2017 and June 2018 in Brazil^10^. Data collection used a complex two-stage cluster sampling plan, with census tracts being selected in the first stage and households in the second. The census tracts come from the master sample of the Brazilian Institute of Geography and Statistics (IBGE), grouped into strata of households with high geographic homogeneity in the sector. Data collection took place throughout 2017 and 2018, divided into four quarters to consider dietary variability and foods at different times of the year^10^.

The POF INA involved 46,164 residents aged ≥10 years in Brazil. The household sample was randomly selected, and all individuals in the target age group were invited to participate. By applying the sampling plan, information was obtained from 52,906,759 Brazilians aged ≥10 years^10^.

### Personal food consumption

The individuals’ food consumption was assessed through two food records applied on two non-consecutive days using the Automated Multiple-Step Method^11^. In several steps, information was collected on all foods consumed on the day before the application, their quantities in household measurements, method of preparation and, for some pre-determined foods, information was requested on the addition of ingredients such as sugars, sweeteners and oils.

Foods with quantities considered unlikely or absent were imputed using the similarity matrix method^12^ based on variables correlated with the possible quantity consumed. The foods were combined with the food codes present in the Brazilian Food Composition Table (TBCA) ^13^, while the preparations were disaggregated considering the standardized TBCA recipes. Finally, the reported/imputed quantity of each food was converted into kilocalories (kcal) using the TBCA information.

Subsequently, the foods were classified according to the NOVA^1^ criteria into FMPF, culinary ingredients, processed foods and UPF. The classification of UPF followed the concept that they are industrial formulations obtained through the fractionation of foods from FMPF^1^.

For this work, the percentage shares of energy provided by UPF and FMPF were considered outcomes, which were obtained through the equation:


$$\%\;of\;energy\;share\;=\frac{\left(kcal\;from\;UPF\;or\;FMPF\;\times 100\right)}{total\;caloric\;consumption\;of\;the\;individual}$$

### Sociodemographic variables

Sociodemographic information was collected through standardized questionnaires to inform residents. The following variables were used: sex (male/female), household situation (urban/rural), region of residence (North, Northeast, South, Southeast and Central-West) and per capita family income based on ¼ of the minimum wage in force in 2018 (<¼/≥¼).

#### Data analysis

Initially, categorical data were described in absolute (n) and relative (%) frequencies. Continuous variables had their normality assumptions tested using the one-sample asymptotic Kolmogorov-Smirnov test, which were rejected when p<0.05, and were therefore described in medians and interquartile ranges (IQR). Since this was a study with complex sampling, sample weights were considered in all analyses.

#### Mixed effects models

To determine the natural heterogeneity among individuals resulting from sociodemographic or environmental factors, mixed linear models were tested. In this regression model, the coefficients (β) are called fixed effects, while the variances and covariances (α) are called variance components. In addition, there is the presence of an individual measure that will differ from the observed mean of the outcome, which is called random error. These random effects will indicate the natural heterogeneity among individuals resulting from the set of observed factors.

In the end, the observed random effects will designate a mathematical measure that will indicate how a set of factors behaves in relation to the observed mean of the outcome. For this study, models containing the following variables were tested: region of residence, household situation, sex and per capita family income. The random effects obtained are the result of the combinatorial analysis of the number of categories contained in each variable inserted in the model, namely, 40 random effects were obtained (5×2×2×2). However, only those that did not include the value 0 in their 95% confidence interval were considered significant random effects. To better represent the effects of sociodemographic factors on the outcomes, dot plots were used.

The analyses were performed in the open access statistical program R (R Core Team, 2023). The linear mixed models were tested using the lme4^14^ and lmer packages, and the sample weights were included using the survey^15^ package for sample expansion.

#### Ethical aspects

The data for this study come from an open access information system, and we therefore dispensed with the requirement for prior request to government agencies or institutions and approval by a research ethics committee.

## RESULTS

### Description of sample

The population assessed stood out for being mostly women (54.1%), with an average age of 39 years, who lived in urban areas (85%) and in the Southeast region of the country (43.2%). Furthermore, the median per capita family income of the individuals was R$899.20 (R$457.60 to R$1609.90) (Table 1). The population's diet was predominantly composed of FMPF (55.3%), with UPF coming in second with 37.5% (Table 1).

**Table 1:** Sociodemographic and food consumption description of Brazilian individuals ≥10 years of age. Brazil, 2017-2018.

Variables		n (%)
Sex		
	Male	24,280,128 (45.9)
	Female	28,626,631 (54.1)
Household situation		
	Urban	44,950,344 (85.0)
	Rural	7,956,415 (15.0)
Region of residence		
	Central-West	4,152,505 (7.6)
	Northeast	14,645,832 (27.0)
	North	3,861,475 (8.2)
	Southeast	22,878,689 (42.7)
	South	7,368,257 (14.5)
Years of study		9.0 (5.0–12.0)*
Age (in years)		39.0 (24.0–55.0)*
Per capita family income		899.2 (457.6–1609.9)*
% of dietary share of FMPF in diet		55.3 (42.0–69.3)*
% of dietary share of UPF in diet		37.5 (24.4–51.6)*
Total		52,906,759 (100.0)

* Median (IQR).

FMPF: fresh or minimally processed foods; UPF: ultra-processed foods.

### Heterogeneity in the consumption of ultra-processed foods

**Figure 1:**
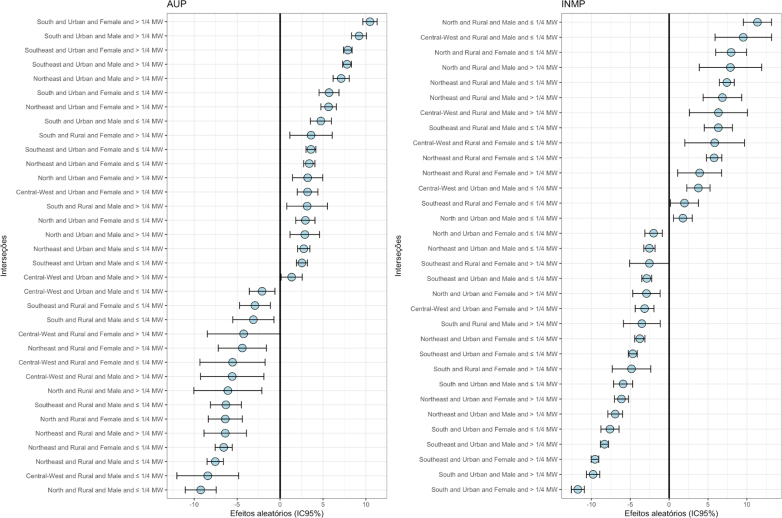
Random effects dot plot on the consumption of natural or minimally processed foods and ultra-processed foods in the Brazilian population ≥10 years of age. Brazil, 2017–2018.

FMPF: fresh or minimally processed; UPF: ultra-processed foods.

Regarding UPF consumption, 34 random effects were significant, with 19 of them (55.9%) driving the average UPF share in the diet of individuals (Figure 1 and Table 2). It is noteworthy that the factors with the greatest driving effect on UPF consumption were similar in that they included conditions such as living in urban areas in the South of the country, followed by the Southeast region, while having a per capita income above ¼ of the minimum wage for both sexes, but still higher for women. Next, living in rural areas of the country appeared only twice in the 19 effects that increased UPF consumption (10.5%), but again associated with the South region with higher income for both sexes. In the factors that reduced UPF consumption (44.1%), there was a strong presence of individuals who lived in rural areas and had lower per capita income, living mainly in the North and Northeast regions (Table 2).

**Table 2:** Random effects on the consumption of ultra-processed foods in Brazilian individuals ≥10 years of age, 2017-2018.

Line No.	UPF			
	Intersections	Random effect	95%CI	
1	South and urban and female and >¼ MW	10.47	9.64	11.30
2	South and urban and male and >¼ MW	9.19	8.34	10.05
3	Southeast and urban and female and >¼ MW	7.89	7.39	8.38
4	Southeast and urban and male and >¼ MW	7.80	7.29	8.32
5	Northeast and urban and male and >¼ MW	7.12	6.18	8.06
6	South and urban and female and ≤¼ MW	5.70	4.54	6.87
7	Northeast and urban and female and >¼ MW	5.65	4.76	6.54
8	South and urban and male and ≤¼ MW	4.75	3.53	5.97
9	South and rural and female and >¼ MW	3.60	1.14	6.07
10	Southeast and urban and female and ≤¼ MW	3.60	3.02	4.17
11	Northeast and urban and female and ≤¼ MW	3.40	2.75	4.06
12	North and urban and female and >¼ MW	3.21	1.46	4.97
13	Central-West and urban and female and >¼ MW	3.20	1.99	4.40
14	South and rural and male and >¼ MW	3.15	0.79	5.52
15	North and urban and female and ≤¼ MW	2.95	1.83	4.07
16	North and urban and male and >¼ MW	2.88	1.16	4.60
17	Northeast and urban and male and ≤¼ MW	2.76	2.05	3.48
18	Southeast and urban and male and ≤¼ MW	2.54	1.91	3.18
19	Central-West and urban and male and >¼ MW	1.35	0.12	2.57
20	Central-West and urban and male and ≤¼ MW	-2.10	-3.59	-0.60
21	Southeast and rural and female and ≤¼ MW	-2.93	-4.72	-1.13
22	South and rural and male and ≤¼ MW	-3.11	-5.51	-0.72
23	Central-West and rural and female and >¼ MW	-4.25	-8.48	-0.01
24	Northeast and rural and female and >¼ MW	-4.40	-7.20	-1.60
25	Central-West and rural and female and ≤¼ MW	-5.54	-9.34	-1.74
26	Central-West and rural and male and >¼ MW	-5.58	-9.27	-1.89
27	North and rural and male and >¼ MW	-6.08	-10.05	-2.12
28	Southeast and rural and male and ≤¼ MW	-6.32	-8.12	-4.51
29	North and rural and female and ≤¼ MW	-6.39	-8.37	-4.41
30	Northeast and rural and male and >¼ MW	-6.39	-8.87	-3.92
31	Northeast and rural and female and ≤¼ MW	-6.57	-7.56	-5.58
32	Northeast and rural and male and ≤¼ MW	-7.55	-8.51	-6.60
33	Central-West and rural and male and ≤¼ MW	-8.42	-12.02	-4.82
34	North and rural and male and ≤¼ MW	-9.24	-11.04	-7.44

Random effect: addition or subtraction on the population mean of the outcome consumption of ultra-processed foods; UPF: ultra-processed foods; 95%CI: 95% confidence interval; ¼ MW: 25% of the minimum wage in 2018 (R$ 238.50 of R$ 954).

### Heterogeneity in the consumption of fresh or minimally processed foods

Regarding the consumption of FMPF, 32 significant random effects were observed, with 14 of them (43.8%) driving its average share (Figure 1 and Table 3). In this food group, the strong presence of rural areas can be highlighted, as well as living in the North, Northeast and Central-West regions, and having a low income. Furthermore, the absence of the South of the country in any effect is noted.

**Table 3:** Random effects on the consumption of fresh or minimally processed foods in Brazilian individuals ≥10 years of age,. Brazil, 2017-2018.

Line No.	FMPF			
	Intersections	Random effect	95%CI	
1	North and rural and male and ≤¼ MW	11.35	9.55	13.15
2	Central-West and rural and male and ≤¼ MW	9.53	5.90	13.16
3	North and rural and female and ≤¼ MW	7.96	5.98	9.95
4	North and rural and male and >¼ MW	7.88	3.87	11.89
5	Northeast and rural and male and ≤¼ MW	7.42	6.47	8.37
6	Northeast and rural and male and >¼ MW	6.85	4.37	9.33
7	Central-West and rural and male and >¼ MW	6.33	2.61	10.06
8	Southeast and rural and male and ≤¼ MW	6.32	4.52	8.12
9	Central-West and rural and female and ≤¼ MW	5.86	2.02	9.69
10	Northeast and rural and female and ≤¼ MW	5.78	4.79	6.77
11	Northeast and rural and female and >¼ MW	3.92	1.10	6.74
12	Central-West and urban and male and ≤¼ MW	3.76	2.27	5.25
13	Southeast and rural and female and ≤¼ MW	1.97	0.17	3.77
14	North and urban and male and ≤¼ MW	1.76	0.56	2.97
15	North and urban and female and ≤¼ MW	-2.00	-3.12	-0.88
16	Northeast and urban and male and ≤¼ MW	-2.54	-3.25	-1.82
17	Southeast and rural and female and >¼ MW	-2.54	-5.08	0.00
18	Southeast and urban and male and ≤¼ MW	-2.88	-3.52	-2.25
19	North and urban and female and >¼ MW	-2.92	-4.68	-1.16
20	Central-West and urban and female and >¼ MW	-3.16	-4.36	-1.95
21	South and rural and male and >¼ MW	-3.51	-5.88	-1.14
22	Northeast and urban and female and ≤¼ MW	-3.77	-4.43	-3.12
23	Southeast and urban and female and ≤¼ MW	-4.67	-5.25	-4.10
24	South and rural and female and >¼ MW	-4.84	-7.31	-2.37
25	South and urban and male and ≤¼ MW	-5.92	-7.14	-4.70
26	Northeast and urban and female and >¼ MW	-6.13	-7.02	-5.24
27	Northeast and urban and male and >¼ MW	-6.93	-7.88	-5.99
28	South and urban and female and ≤¼ MW	-7.61	-8.78	-6.45
29	Southeast and urban and male and >¼ MW	-8.31	-8.83	-7.80
30	Southeast and urban and female and >¼ MW	-9.53	-10.03	-9.04
31	South and urban and male and >¼ MW	-9.78	-10.63	-8.92
32	South and urban and female and >¼ MW	-11.75	-12.58	-10.92

Random effect: addition or subtraction on the population mean of the outcome consumption of ultra-processed foods; FMPF: fresh minimally processed foods; 95%CI: 95% confidence interval; ¼ MW: 25% of the minimum wage in 2018 (R$ 238.50 of R$ 954).

Regarding the factors that reduced the consumption of FMPF (56.2%), the most prevalent factors identified were living in urban areas, especially in the South and Southeast regions, as well as having a higher family income, diametrically opposed factors on the consumption of UPF (Table 3).

## DISCUSSION

The consumption of UPF among the individuals evaluated was high and quite heterogeneous. All geographic macro-regions were found to be drivers of UPF consumption, but the South and Southeast stood out, as well as the majority being located in urban areas and having an income above ¼ of the minimum wage. Such factors may be associated with the provision of conditions that facilitate access to these food products. In contrast, FMPF foods were found in the North, Northeast and Central-West regions, especially in low-income strata and rural areas, which may possibly indicate cultivation environments and greater access to these foods.

In addition, it is noteworthy that the consumption of FMPF was over half of the energy consumption of the Brazilian population, which can be explained by the Western or traditional Brazilian dietary patterns, which are composed mainly of the consumption of bread, coffee, rice, beans, animal protein and some salad option^16–19^. However, when observing the consumption of UPF, a high dietary participation is noted, being almost a third of the total usual intake of the population. It is important to note that there is still no upper limit for UPF consumption, but it is suggested that its dietary contribution be as low as possible^20^.

Studies indicate that there is significant heterogeneity in the consumption of FMPF and UPF in different populations around the world, with the highest consumption of the latter concentrated in urban and more developed areas^4,6,7,21–24^. Previous findings from the POF itself reinforce that the participation of UPF in the diet of Brazilians is strongly related to the place of residence, with states from the South and Southeast regions being those with the highest consumption, as well as the highest level of education, age and household situation^3^.

Despite the recognized differences in food consumption patterns caused by the age of individuals^6,7,22^, in this study this variable was not added to the mixed linear model, as it did not show a significant effect on consumption when considering the different intersections with the other sociodemographic factors.

The effects of these factors on the consumption of UPF are widely studied singly; however, it is important to highlight that there are different degrees of magnitude on consumption when considering the intersection between one or more sociodemographic conditions. The effects of living in urban or rural areas, for example, are recognized as distinct on the consumption of UPF or FMPF, since conditions such as the ability to penetrate more remote areas and a strong presence of agricultural products in rural areas are considered^3,25,26^. However, this study identified that in the southern region of Brazil, regardless of being a rural area, the participation of UPF in the diet of individuals was still higher than the national average. This may have been due to the greater development of the region compared to other areas of the country, as well as issues related to access and purchasing power^18,27,28^.

It is worth noting that higher income is a factor that may be associated with guaranteed access to ultra-processed products^29–31^, which, together with living in regions with low penetration of these products, such as rural areas, justifies the strong reduction in the participation of these products^32^.

Regarding the participation of FMPF in the diet, it was observed that its consumption was dictated by access conditions. The greatest consumption of these products was mainly in rural regions, which are usually agricultural centers with easier access to fresh food and, usually, with lower prices, as well as the geographic regions that appeared are the least industrialized^25,32^. Thus, it is suggested that the consumption of FMPF, in addition to taking into account factors such as geographic proximity and ease of access to these foods, may also be dependent on the lack of competitiveness with a greater variety of UPF^5,33^.

Conditions such as income can also be strong determinants, since the population with the lowest income in these regions may be small rural producers who depend almost exclusively on their own crops^25,32^. Furthermore, despite the strong effect of rural household conditions on the consumption of FMPF, it is important to emphasize that even in these areas, rural populations in the Southeast and South had lower averages of these foods, possibly due to access issues previously elucidated in this study, as well as industrialization^3,34,35^.

The role of sex in food consumption is quite heterogeneous in the literature. Some authors point out that women are usually more concerned about their health status and therefore have lower consumption of UPF^6,24,31^. This role is controversial, since some classes of UPF may have more overt advertising targeting the female public and are not considered unhealthy^3^. However, strong heterogeneity was observed, in which there was no emphasis on higher or lower consumption of UPF or FMPF in either sex. It is suggested that the conditions of each individual's immediate surroundings have a greater effect on consumption.

Furthermore, it is important to emphasize that the data used in this study are from the 2017-2018 edition of the POF. However, it is possible to expect that after the COVID-19 pandemic, the increase in UPF consumption in Brazil has continued, especially in large centers, due to social isolation and the preference for ready-to-eat foods^36,37^. However, it is possible that in rural regions and regions further away from urban centers the preference for FMPF foods has remained, making it possible to maintain the panorama of heterogeneity observed.

The strengths of this study include a nationally representative probabilistic sample and the use of a robust statistical analysis that promotes the interaction of different sociodemographic conditions and their effects on the dietary participation of UPF and FMPF in the diet of the Brazilian population. The mixed-effects regression models used allow for a more flexible approach when compared to traditional regression models. Since traditional models use only explanatory variables and their effect on an outcome, mixed-effects models include both fixed and random parameters, allowing the estimation of variability between individuals or groups^38^. By including random effects, mixed-effects models capture the correlation between observations within the same groups or individuals, resulting in more accurate estimates of population parameters and better reflecting the variability between groups^38^.

Furthermore, this study is the first to assess the consumption of FMPF and UPF from a nationally representative sample using robust analyses such as mixed linear models. The use of this analysis allows the identification of consumption patterns of both food groups, considering the intersection of a set of sociodemographic factors. In this way, it is possible to observe with greater clarity and depth the dynamics of consumption of these foods through different factors that make up the immediate environment of different Brazilian population groups.

This method allows the observation of different combinations of factors that lead to greater or lesser participation compared to the usual observation of isolated associated factors. However, limitations may arise from potential overestimations or underestimations of some food groups and differences between culinary recipes compared to what is standardized in food composition tables. However, to minimize these biases, validated food survey methods were used, as well as all culinary preparations were broken down to obtain the isolated composition of each ingredient that composes it.

Finally, this study provides a strong contribution to the national and international literature by describing the effects of different sociodemographic conditions on the dietary participation of UPF and FMPF in the Brazilian population aged ≥10 years. Widely recognized conditions on UPF and FMPF consumption were observed and confirmed, but different mechanisms of interaction between them were understood, reinforcing the consumption of these food groups as dependent on access conditions. The heterogeneity in UPF and FMPF consumption could be perceived as highly dependent on conditions that favor or hinder access to these foods, especially sociodemographic characteristics such as income and place of residence. Furthermore, through the results of this work, understanding the focal groups most exposed to the consumption of UPF and its future harmful effects, as well as lower consumption of FMPF, will allow food and nutrition departments and coordinators to understand the effects of existing food and nutrition surveillance actions and be able to target specific population segments to mitigate the consumption of UPF or encourage the consumption of FMPF.
