# Supplementary material for: Heterogeneity in the consumption of fresh and ultra-processed foods by the Brazilian population ≥10 years of age
Source: Rev Bras Epidemiol. 2024 Dec 16;27:e240069. doi: 10.1590/1980-549720240069 (PMC11654637; doi:10.1590/1980-549720240069)
Supplement: Translation

# Translation: Heterogeneidade no consumo de alimentos *in natura* e ultraprocessados da população Brasileira ≥10 anos de idade

**DOI:** 10.1590/1980-549720240069.2

**Authors:** Victor Nogueira da Cruz Silveira, Ana Karina Teixeira da Cunha França, Alcione Miranda dos Santos

**Affiliations:** I Universidade Federal do Maranhão, Programa de Pós-Graduação em Saúde Coletiva – São Luís (MA), Brasil.

**Keywords:** Alimento processado, Dieta, Brasil, Consumo alimentar, Inquéritos sobre dietas

## Abstract

**Objetivo:**

Avaliar a heterogeneidade no consumo de alimentos *in natura* ou minimamente processados (INMP) e alimentos ultraprocessados (AUP) da população brasileira ≥10 anos de idade.

**Métodos:**

Estudo transversal que utilizou dados do Inquérito Nacional de Alimentação e do módulo do morador da edição de 2017–2018 da Pesquisa de Orçamentos Familiares. Foram utilizadas as variáveis relativas ao sexo, à região de residência, situação domiciliar e renda familiar per capita em salários-mínimos. Os desfechos foram a participação dietética em percentual de INMP e AUP. A heterogeneidade foi avaliada por meio dos efeitos aleatórios (EA) produzidos por modelos lineares de efeitos mistos.

**Resultados:**

Foram obtidos 32 EA para o consumo de INMP e 34 para os AUP. Residir na zona urbana das regiões Sul e Sudeste bem como ter maior renda foram fatores impulsionadores no consumo de AUP e redutores no consumo de INMP. Residir na zona rural e ter baixa renda foram principalmente fatores redutores no consumo de AUP e impulsionadores no consumo de INMP.

**Conclusões:**

O consumo de AUP e INMP apresentaram forte heterogeneidade com bastante efeitos aleatórios encontrados para ambos. Fatores como renda e situação domiciliar foram os principais marcadores no consumo alimentar dos indivíduos.

## INTRODUÇÃO

Alimentos *in natura* ou minimamente processados (INMP) são aqueles diretamente obtidos de plantas ou animais que não perpassaram por alterações após deixar a natureza, ou sofreram alterações mínimas^1^. Eles são caracterizados por não conterem aditivos químicos, logo tornando-se a base para uma alimentação saudável^2^. Em oposição, os alimentos ultraprocessados (AUP) são produtos industriais feitos com base em INMP ou sintetizados em laboratório, que possuem adição de compostos químicos que alteram suas propriedades sensoriais^1^.

Esses alimentos diferenciam-se em função de seu grau de processamento, bem como na adição de substâncias artificiais^1–3^. O uso desses componentes industriais garante aos AUP maior vida de prateleira, palatabilidade e alguma similaridade com os INMP aos quais eles fazem referência^1^.

O consumo de AUP tornou-se uma preocupação global, uma vez que sua ingestão está associada a diversos efeitos deletérios em saúde^4^. Porém, apesar de reconhecidamente prejudiciais à saúde, seu consumo apresenta tendência crescente, possivelmente decorrente da praticidade e hiperpalatabilidade que ofertam^3,5^.

No Brasil, o consumo de AUP apresenta padrão diverso, sendo maior em mulheres, nas regiões Sul e Sudeste, bem como em populações com maior renda e escolaridade, porém com forte divergência sobre as classes de ultraprocessados consumidas^3,6,7^. Ainda, apesar da tendência de decréscimo global no consumo desses alimentos, o Brasil enfrenta uma tendência de progressão possivelmente provocada por marketing ostensivo e praticidade no consumo desses produtos^8^. Em oposição, populações com menor poder aquisitivo, racializadas e habitantes da zona rural tendem a consumir menos dos referidos produtos industriais e possuir uma dieta rica em INMP^3,8^.

Diferentes estudos têm evidenciado os efeitos sociodemográficos sobre o consumo alimentar no Brasil, e usualmente utilizam modelos de regressão lineares ou generalizados para testar suas hipóteses^4^. Porém, o consumo alimentar é um comportamento multimotivado que pode ser modificado mediante a combinação de um conjunto de fatores, e não apenas um isoladamente. Neste sentido, os modelos de regressão de efeitos mistos surgem como ferramentas estatísticas poderosas para considerar a variação entre indivíduos e grupos^9^.

Na avaliação do consumo de alimentos ultraprocessados, esses modelos podem levar em conta fatores individuais — como idade, sexo, escolaridade — e contextuais — como região geográfica e renda — para entender melhor as associações e prever mudanças ao longo do tempo. Assim, compreender as diferenças nos padrões de consumo de alimentos ultraprocessados é crucial para o entendimento e direcionamento estratégico de políticas de prevenção e promoção da saúde para um setor específico a fim de mitigar o consumo desses alimentos, bem como prevenir seus efeitos deletérios. Ao considerar fatores socioeconômicos e demográficos, intervenções específicas para grupos de maior risco podem ser planejadas e aplicadas.

Dessa forma, este trabalho possui como objetivo avaliar a heterogeneidade no consumo de alimentos *in natura* ou minimamente processados (INMP) e alimentos ultraprocessados (AUP) da população brasileira ≥10 anos de idade.

## MÉTODOS

### Desenho e amostra

Trata-se de um estudo transversal com dados do módulo de consumo alimentar pessoal do Inquérito Nacional de Alimentação (INA) da Pesquisa de Orçamentos Familiares (POF) — pesquisa de representatividade nacional realizada entre julho de 2017 e junho de 2018 no Brasil^10^. A coleta de dados utilizou um plano amostral complexo por conglomerados em dois estágios, com sorteio de setores censitários no primeiro estágio e domicílios no segundo. Os setores censitários são oriundos da amostra mestra do Instituto Brasileiro de Geografia e Estatística (IBGE), agrupados em estratos de domicílios com elevada homogeneidade geográfica no setor. A coleta de dados ocorreu ao longo dos anos de 2017 e 2018, dividida em quatro trimestres para considerar a variabilidade alimentar e os alimentos das diferentes épocas do ano^10^.

O INA da POF envolveu 46.164 residentes com idade ≥10 anos no Brasil. A amostra de domicílios foi selecionada aleatoriamente, e todos os indivíduos da faixa etária alvo foram convidados a participar. Com a aplicação do plano amostral, foram obtidas informações de 52.906.759 brasileiros com idade ≥10 anos^10^.

### Consumo alimentar pessoal

O consumo alimentar dos indivíduos foi avaliado por meio de dois registros alimentares aplicados em dois dias não consecutivos pelo Método Automatizado de Múltiplas Etapas^11^. Em diversas etapas, foram coletadas informações sobre todos os alimentos consumidos no dia anterior à aplicação, suas quantidades em medidas caseiras, modo de preparo e, para alguns alimentos pré-determinados, foram solicitadas informações sobre a adição de ingredientes como açúcares, adoçantes e óleos.

Os alimentos com quantidades consideradas improváveis ou ausentes foram imputados pelo método da matriz de similaridade^12^ a partir de variáveis correlacionadas com a possível quantidade consumida. Os alimentos foram combinados com os códigos alimentares presentes na Tabela Brasileira de Composição de Alimentos (TBCA)^13^, enquanto as preparações foram desagregadas considerando as receitas padronizadas da TBCA. Por fim, a quantidade informada/imputada de cada alimento foi convertida em quilocalorias (kcal) utilizando as informações do TBCA.

Posteriormente, os alimentos foram classificados, segundo os critérios de classificação NOVA^1^, em alimentos *in natura* ou minimamente processados (INMP), ingredientes culinários, alimentos processados e alimentos ultraprocessados (AUP). A classificação dos AUP seguiu o conceito de que são formulações industriais obtidas através do fracionamento de alimentos provenientes de alimentos INMP^1^.

Para este trabalho, foram considerados desfechos as participações percentuais de energia fornecidas pelos AUP e INMP, as quais foram obtidos por meio da equação:


$$\%\;de\;participa\text{çã}o\;energ\text{é}tica=\frac{\left(kcal\;proveniente\;de\;AUP\;ou\;INMP\times 100\right)}{consumo\;cal\text{ó}rico\;total\;do\;indiv\text{í}duo}$$

### Variáveis sociodemográficas

As informações sociodemográficas foram coletadas por meio de questionários padronizados de informações aos residentes. Foram utilizadas as seguintes variáveis: sexo (masculino/feminino), situação domiciliar (urbana/rural), região de residência (Norte, Nordeste, Sul, Sudeste e Centro-Oeste) e renda familiar per capita mediante ¼ de salário-mínimo vigente em 2018 (<¼/≥¼).

#### Análise de dados

Inicialmente, os dados categóricos foram descritos em frequências absolutas (n) e relativas (%). As variáveis contínuas tiveram seus pressupostos de normalidade testados por meio do teste assintótico uniamostral de Kolmogorov-Smirnov, os quais foram rejeitados quando p<0,05, sendo, portanto, descritos em medianas e intervalos interquartis (IQR). Por se tratar de estudo com amostragem complexa, os pesos amostrais foram considerados em todas as análises.

#### Modelos de efeitos mistos

Para determinar a heterogeneidade natural entre os indivíduos decorrente de fatores sociodemográficos ou ambientais, foram testados modelos lineares mistos. Neste modelo de regressão, os coeficientes (β) são denominados como efeitos fixos, enquanto as variâncias e covariâncias (α) são denominadas como componentes da variância. Ademais, há a presença de uma medida individual que irá diferir da média observada do desfecho, que é denominada de erro aleatório. Esses efeitos aleatórios irão indicar a heterogeneidade natural entre os indivíduos decorrente do conjunto de fatores observados.

Ao final, os efeitos aleatórios observados irão designar uma medida matemática que indicará como um conjunto de fatores comporta-se em relação à média observada do desfecho. Para este trabalho, foram testados modelos contendo as seguintes variáveis: região de residência, situação domiciliar, sexo e renda familiar per capita. Os efeitos aleatórios obtidos são resultantes da análise combinatória do número de categorias contidas em cada variável inserida no modelo, a saber, foram obtidos 40 efeitos aleatórios (5×2×2×2). No entanto, foram considerados como efeitos aleatórios significativos apenas aqueles que não incluem o valor 0 (zero) em seu intervalo de confiança de 95%. Para melhor representação dos efeitos de fatores sociodemográficos sobre os desfechos, foram utilizados gráficos de pontos.

As análises foram realizadas no programa estatístico de acesso aberto R (*R Core Team*, 2023). Os modelos lineares mistos foram testados por meio dos pacotes lme4^14^ e lmer, e os pesos amostrais foram incluídos por meio do pacote *survey* ^15^ para expansão amostral.

#### Aspectos éticos

Os dados deste estudo são oriundos de um sistema de informação de acesso aberto, dispensando, portanto, a obrigatoriedade de solicitação prévia a órgãos ou instituições governamentais e aprovação em Comitê de Ética em Pesquisa.

## RESULTADOS

### Descrição da amostra

A população avaliada destacou-se por constituir-se, em sua maioria, de mulheres (54,1%), com idade média de 39 anos, que residiam na zona urbana (85%) e na região Sudeste do país (43,2%). Ainda, a renda familiar per capita mediana dos indivíduos foi de R$ 899,20 (R$ 457,60 a R$ 1609,90) (Tabela 1). A dieta da população era predominantemente composta por alimentos *in natura* ou minimamente processados (55,3%), com alimentos ultraprocessados figurando logo após com 37,5% (Tabela 1).

**Tabela 1:** Descrição sociodemográfica e do consumo alimentar de indivíduos brasileiros ≥ 10 anos de idade. Brasil, 2017-2018.

Variáveis		n (%)
Sexo		
	Masculino	24.280.128 (45,9)
	Feminino	28.626.631 (54,1)
Situação domiciliar		
	Urbana	44.950.344 (85,0)
	Rural	7.956.415 (15,0)
Região de residência		
	Centro-Oeste	4.152.505 (7,6)
	Nordeste	14.645.832 (27,0)
	Norte	3.861.475 (8,2)
	Sudeste	22.878.689 (42,7)
	Sul	7.368.257 (14,5)
Anos de estudo		9,0 (5,0–12,0)*
Idade (em anos)		39,0 (24,0–55,0)*
Renda familiar per capita		899,2 (457,6–1609,9)*
% de participação dietética de INMP na dieta		55,3 (42,0–69,3)*
% de participação dietética de AUP na dieta		37,5 (24,4–51,6)*
Total		52.906.759 (100,0)

* Mediana (IIQ).

INMP: *in natura* ou minimamente processados; AUP: alimentos ultraprocessados.

### Heterogeneidade no consumo de alimentos ultraprocessados

Acerca do consumo de AUP, 34 efeitos aleatórios foram significantes, sendo 19 deles (55,9%) impulsionadores na participação média de AUP na dieta dos indivíduos (Figura 1 e Tabela 2). Destaca-se que os fatores com maior impulsionamento no consumo de AUP assemelham-se por conterem condições como residir em áreas urbanas da região Sul do país, seguidamente da região Sudeste, enquanto possui renda per capita superior a ¼ de salário-mínimo em ambos os sexos, porém ainda superior no público feminino. Seguidamente, residir na área rural do país figurou apenas duas vezes nos 19 efeitos que incrementam o consumo de AUP (10,5%), porém novamente associada à região Sul com maior renda em ambos os sexos. Nos fatores redutores do consumo de AUP (44,1%), observa-se uma forte presença de indivíduos que residem na zona rural e com renda per capita inferior, com residência sobretudo nas regiões Norte e Nordeste (Tabela 2).

**Figura 1:** Gráfico de pontos de efeitos aleatórios sobre o consumo de alimentos *in natura* ou minimamente processados e alimentos ultraprocessados na população brasileira ≥10 anos de idade. Brasil, 2017–2018.

INMP: *in natura* ou minimamente processados; AUP: alimentos ultraprocessados.

**Tabela 2:** Efeitos aleatórios sobre o consumo de alimentos ultraprocessados em indivíduos brasileiros ≥10 anos de idade. Brasil, 2017-2018.

N^o^ de linha	AUP			
	Interseções	Efeito aleatório	IC95%	
1	Sul e urbano e feminino e >¼ SM	10.47	9.64	11.30
2	Sul e urbano e masculino e >¼ SM	9.19	8.34	10.05
3	Sudeste e urbano e feminino e >¼ SM	7.89	7.39	8.38
4	Sudeste e urbano e masculino e >¼ SM	7.80	7.29	8.32
5	Nordeste e urbano e masculino e >¼ SM	7.12	6.18	8.06
6	Sul e urbano e feminino e ≤¼ SM	5.70	4.54	6.87
7	Nordeste e urbano e feminino e >¼ SM	5.65	4.76	6.54
8	Sul e urbano e masculino e ≤¼ SM	4.75	3.53	5.97
9	Sul e rural e feminino e >¼ SM	3.60	1.14	6.07
10	Sudeste e urbano e feminino e ≤¼ SM	3.60	3.02	4.17
11	Nordeste e urbano e feminino e ≤¼ SM	3.40	2.75	4.06
12	Norte e urbano e feminino e >¼ SM	3.21	1.46	4.97
13	Centro-Oeste e urbano e feminino e >¼ SM	3.20	1.99	4.40
14	Sul e rural e masculino e >¼ SM	3.15	0.79	5.52
15	Norte e urbano e feminino e ≤¼ SM	2.95	1.83	4.07
16	Norte e urbano e masculino e >¼ SM	2.88	1.16	4.60
17	Nordeste e urbano e masculino e ≤¼ SM	2.76	2.05	3.48
18	Sudeste e urbano e masculino e ≤¼ SM	2.54	1.91	3.18
19	Centro-Oeste e urbano e masculino e >¼ SM	1.35	0.12	2.57
20	Centro-Oeste e urbano e masculino e ≤¼ SM	-2.10	-3.59	-0.60
21	Sudeste e rural e feminino e ≤¼ SM	-2.93	-4.72	-1.13
22	Sul e rural e masculino e ≤¼ SM	-3.11	-5.51	-0.72
23	Centro-Oeste e rural e feminino e >¼ SM	-4.25	-8.48	-0.01
24	Nordeste e rural e feminino e >¼ SM	-4.40	-7.20	-1.60
25	Centro-Oeste e rural e feminino e ≤¼ SM	-5.54	-9.34	-1.74
26	Centro-Oeste e rural e masculino e >¼ SM	-5.58	-9.27	-1.89
27	Norte e rural e masculino e >¼ SM	-6.08	-10.05	-2.12
28	Sudeste e rural e masculino e ≤¼ SM	-6.32	-8.12	-4.51
29	Norte e rural e feminino e ≤¼ SM	-6.39	-8.37	-4.41
30	Nordeste e rural e masculino e >¼ SM	-6.39	-8.87	-3.92
31	Nordeste e rural e feminino e ≤¼ SM	-6.57	-7.56	-5.58
32	Nordeste e rural e masculino e ≤¼ SM	-7.55	-8.51	-6.60
33	Centro-Oeste e rural e masculino e ≤¼ SM	-8.42	-12.02	-4.82
34	Norte e rural e masculino e ≤¼ SM	-9.24	-11.04	-7.44

Efeito aleatório: adição ou subtração sobre a média populacional do desfecho consumo de alimentos ultraprocessados; AUP: alimentos ultraprocessados; IC95%: intervalo de confiança de 95%; ¼ SM: 25% do salário-mínimo vigente em 2018 (R$ 238,50 de R$ 954).

### Heterogeneidade no consumo de in natura ou minimamente processados

Relativo ao consumo de INMP, foram observados 32 efeitos aleatórios significantes, com 14 deles (43,8%) sendo impulsionadores de sua participação média (Figura 1 e Tabela 3). Neste grupo de alimentos, pode ser destacada a forte presença da área rural, bem como residir nas regiões Norte, Nordeste e Centro-Oeste, e possuir baixa renda. Ainda, nota-se a ausência da região Sul do país em qualquer efeito.

**Tabela 3:** Efeitos aleatórios sobre o consumo de alimentos *in natura* ou minimamente processados em indivíduos brasileiros ≥10 anos de idade. Brasil, 2017-2018.

N^o^ de linha	INMP			
	Interseções	Efeito aleatório	IC95%	
1	Norte e rural e masculino e ≤¼ SM	11.35	9.55	13.15
2	Centro-Oeste e rural e masculino e ≤¼ SM	9.53	5.90	13.16
3	Norte e rural e feminino e ≤¼ SM	7.96	5.98	9.95
4	Norte e rural e masculino e >¼ SM	7.88	3.87	11.89
5	Nordeste e rural e masculino e ≤¼ SM	7.42	6.47	8.37
6	Nordeste e rural e masculino e >¼ SM	6.85	4.37	9.33
7	Centro-Oeste e rural e masculino e >¼ SM	6.33	2.61	10.06
8	Sudeste e rural e masculino e ≤¼ SM	6.32	4.52	8.12
9	Centro-Oeste e rural e feminino e ≤¼ SM	5.86	2.02	9.69
10	Nordeste e rural e feminino e ≤¼ SM	5.78	4.79	6.77
11	Nordeste e rural e feminino e >¼ SM	3.92	1.10	6.74
12	Centro-Oeste e urbano e masculino e ≤¼ SM	3.76	2.27	5.25
13	Sudeste e rural e feminino e ≤¼ SM	1.97	0.17	3.77
14	Norte e urbano e masculino e ≤¼ SM	1.76	0.56	2.97
15	Norte e urbano e feminino e ≤¼ SM	-2.00	-3.12	-0.88
16	Nordeste e urbano e masculino e ≤¼ SM	-2.54	-3.25	-1.82
17	Sudeste e rural e feminino e >¼ SM	-2.54	-5.08	0.00
18	Sudeste e urbano e masculino e ≤¼ SM	-2.88	-3.52	-2.25
19	Norte e urbano e feminino e >¼ SM	-2.92	-4.68	-1.16
20	Centro-Oeste e urbano e feminino e >¼ SM	-3.16	-4.36	-1.95
21	Sul e rural e masculino e >¼ SM	-3.51	-5.88	-1.14
22	Nordeste e urbano e feminino e ≤¼ SM	-3.77	-4.43	-3.12
23	Sudeste e urbano e feminino e ≤¼ SM	-4.67	-5.25	-4.10
24	Sul e rural e feminino e >¼ SM	-4.84	-7.31	-2.37
25	Sul e urbano e masculino e ≤¼ SM	-5.92	-7.14	-4.70
26	Nordeste e urbano e feminino e >¼ SM	-6.13	-7.02	-5.24
27	Nordeste e urbano e masculino e >¼ SM	-6.93	-7.88	-5.99
28	Sul e urbano e feminino e ≤¼ SM	-7.61	-8.78	-6.45
29	Sudeste e urbano e masculino e >¼ SM	-8.31	-8.83	-7.80
30	Sudeste e urbano e feminino e >¼ SM	-9.53	-10.03	-9.04
31	Sul e urbano e masculino e >¼ SM	-9.78	-10.63	-8.92
32	Sul e urbano e feminino e >¼ SM	-11.75	-12.58	-10.92

Efeito aleatório: adição ou subtração sobre a média populacional do desfecho consumo de alimentos ultraprocessados; INMP: *in natura* ou minimamente processados; IC95%: intervalo de confiança de 95%; ¼ SM: 25% do salário-mínimo vigente em 2018 (R$ 238,50 de R$ 954).

Acerca dos fatores que reduziam o consumo de INMP (56,2%), foram identificados prevalentemente residir em zona urbana, sobretudo nas regiões Sul e Sudeste, bem como possuir maior renda familiar. Fatores diametralmente opostos sobre o consumo de AUP (Tabela 3).

## DISCUSSÃO

O consumo de AUP nos indivíduos avaliados foi elevado com bastante heterogeneidade. Todas as macrorregiões geográficas figuraram nos efeitos impulsionadores do consumo de AUP, porém destacaram-se o Sul e Sudeste, bem como majoritariamente estar localizado em áreas urbanas e possuir renda superior a ¼ de salário-mínimo. Tais fatores podem estar associados ao fornecimento de condições que facilitem o acesso a esses produtos alimentícios. Em oposição, os alimentos INMP figuraram nas regiões Norte, Nordeste e Centro-Oeste, sobretudo em estratos de baixa renda e na zona rural, o que possivelmente pode indicar ambientes de cultivo e maior acesso desses alimentos.

Ademais, ressalta-se que o consumo de INMP foi acima da metade do consumo energético da população brasileira, o que pode ser explicado pelos padrões alimentares ocidentais ou tradicionais brasileiros que são compostos majoritariamente pelo consumo de pães, café, arroz, feijão, proteína animal e alguma opção de salada^16–19^. No entanto, ao observar o consumo de AUP, nota-se elevada participação dietética, sendo quase um terço da ingestão total usual da população. Destaca-se que ainda não se possui um limite superior de consumo de AUP, porém é sugerido que sua participação dietética seja a menor possível^20^.

Estudos indicam que há forte heterogeneidade sobre o consumo de INMP e AUP em distintas populações ao redor do globo, com o maior consumo destes últimos concentrados em zonas urbanas e mais desenvolvidas^4,6,7,21–24^. Achados anteriores da própria POF reforçam que a participação de AUP na alimentação de brasileiros possui forte relação com o local de residência, sendo os estados provenientes das regiões Sul e Sudeste aqueles com maior consumo, assim como a maior escolaridade, idade e situação domiciliar^3^.

Apesar das reconhecidas diferenças nos padrões de consumo alimentar provocados pela idade dos indivíduos^6,7,22^, neste trabalho essa variável não foi adicionada ao modelo linear misto, por não ter apresentado efeito significativo sobre o consumo ao considerar-se as diferentes intercessões com os demais fatores sociodemográficos.

Os efeitos desses fatores sobre o consumo de AUP são amplamente estudados isoladamente, porém, é importante destacar que existem distintos graus de magnitude sobre o consumo ao considerar-se a interseção entre uma ou mais condições sociodemográficas. Os efeitos de residir na região urbana ou rural, por exemplo, são reconhecidamente distintos sobre o consumo de AUP ou INMP, uma vez que são consideradas condições como capacidade de penetração em zonas mais remotas e forte presença de produtos da agricultura em regiões rurais^3,25,26^. No entanto, neste trabalho foi identificado que na região Sul brasileira, independentemente de ser zona rural, a participação de AUP na dieta dos indivíduos ainda era superior à média nacional. Isso pode ter decorrido do maior desenvolvimento da região frente a outras áreas do país, bem como a questões ligadas a acesso e poder aquisitivo^18,27,28^.

Cabe ressaltar que a maior renda é um fator que pode estar associado com a garantia de acesso a produtos ultraprocessados^29–31^, o que, conjuntamente a residir em regiões com baixa penetração desses produtos, tais como zonas rurais, justifica a forte redução sobre a participação desses produtos^32^.

Acerca da participação de INMP na dieta, pôde ser observado que seu consumo foi ditado por condições de acesso. O maior consumo desses produtos foi sobretudo em regiões rurais, que são usualmente polos agriculturais com acesso mais facilitado a alimentos frescos e, usualmente, com menores preços, bem como as regiões geográficas que figuraram são as menos industrializadas^25,32^. Dessa forma, sugere-se que o consumo de INMP, além de levar em consideração fatores como proximidade geográfica e facilidade de acesso a esses alimentos, também pode ser dependente da falta de competitividade com uma maior variedade de AUP^5,33^.

Condições como a renda também podem ser fortes determinantes, uma vez que a população com menor rendimento nessas regiões pode se tratar de pequenos produtores rurais que dependem quase que exclusivamente de seus próprios cultivos^25,32^. Ainda, apesar do forte efeito da situação domiciliar rural sobre o consumo de alimentos INMP, é importante reforçar que mesmo nessas zonas, populações rurais do Sudeste e Sul possuíam médias de participação desses alimentos inferiores, possivelmente por questões de acesso previamente elucidados neste trabalho, bem como industrialização^3,34,35^.

O papel do sexo dos indivíduos no consumo alimentar é bastante heterogêneo na literatura. Alguns autores destacam que mulheres possuem maior preocupação usual com seu estado de saúde e, portanto, possuem menor consumo de AUP^6,24,31^. Esse papel é controverso, uma vez que algumas classes de ultraprocessados podem contar com publicidade mais ostensiva ao público feminino e não serem consideradas não-saudáveis^3^. No entanto, foi observada forte heterogeneidade, em que não há destaque ao maior ou menor consumo de AUP ou INMP em ambos os sexos. Sugere-se que as condições do entorno imediato de cada indivíduo possuam efeito maior sobre o consumo.

Ademais, é importante ressaltar que os dados utilizados neste estudo são da edição de 2017-2018 da POF. Porém, é possível esperar que após a pandemia de COVID-19, a progressão no consumo de AUP no Brasil tenha permanecido, sobretudo em grandes centros, em virtude do isolamento social e da preferência por alimentos prontos para consumo^36,37^. No entanto, é possível que em regiões rurais e mais afastadas de centros urbanos tenha permanecido a preferência pelos alimentos INMP, sendo possível a manutenção do panorama de heterogeneidade observado.

Este estudo possui como pontos fortes uma amostra probabilística de representatividade nacional e o uso de uma análise estatística robusta que promove a interação de distintas condições sociodemográficas e seus efeitos sobre a participação dietética de AUP e INMP na alimentação da população brasileira. Os modelos de regressão de efeitos mistos utilizados permitem uma abordagem mais flexível quando comparados aos modelos de regressão tradicionais. Uma vez que os modelos tradicionais utilizam somente variáveis explicativas e seu efeito sobre um desfecho, os modelos de efeitos mistos incluem tanto parâmetros fixos quanto aleatórios, permitindo a estimativa de variabilidade entre indivíduos ou grupos^38^. Ao incluir efeitos aleatórios, os modelos de efeitos mistos capturam a correlação entre observações dentro dos mesmos grupos ou indivíduos, resultando em estimativas mais precisas dos parâmetros populacionais e refletindo melhor a variabilidade entre os grupos^38^.

Ademais, este trabalho é o primeiro a avaliar o consumo de INMP e AUP proveniente de uma amostra representativa nacional com análises robustas como os modelos lineares mistos. O uso desta análise proporciona a identificação de padrões de consumo de ambos os grupos de alimentos, considerando a intercessão de um conjunto de fatores sociodemográficos. Dessa forma, é possível observar com maior clareza e profundidade a dinâmica do consumo desses alimentos mediante diferentes fatores que compõem o entorno imediato de distintos grupos populacionais brasileiros.

Tal método permite observar diferentes combinações de fatores que levem a maior ou menor participação em comparação à observação usual de fatores associados isolados. No entanto, como limitantes podem advir potenciais superestimações ou subestimações de alguns grupos alimentares e diferenças entre receitas culinárias frente ao que é padronizado em tabelas de composição de alimentos. Porém, como forma de minimizar esses vieses, foram utilizados métodos de inquérito alimentar validados, bem como destrinchadas todas as preparações culinárias, de modo a se obter a composição isolada de cada ingrediente que a compõe.

Finalmente, este trabalho surge como uma forte contribuição à literatura nacional e internacional por descrever os efeitos de diferentes agrupamentos de condições sociodemográficas sobre a participação dietética de AUP e INMP na população brasileira ≥10 anos de idade. Condições largamente reconhecidas sobre o consumo de AUP e INMP foram observadas e confirmadas, porém foram entendidos diferentes mecanismos de interação destas, reforçando o consumo dos referidos grupos de alimentos como dependentes de condições de acesso. A heterogeneidade no consumo de AUP e INMP pôde ser percebida como bastante dependente de condições que favoreçam ou impeçam o acesso a esses alimentos, sobretudo características sociodemográficas como renda e local de residência. Ainda, por meio dos resultados deste trabalho, o entendimento dos grupos focais mais expostos ao consumo de AUP e seus efeitos deletérios futuros, bem como menor consumo de INMP, permitirá que secretarias e coordenações de alimentação e nutrição compreendam os efeitos das ações de vigilância alimentar e nutricional existentes e consigam direcionar sobre os segmentos populacionais específicos de modo a mitigar o consumo de AUP ou incentivar o consumo de INMP.
